# Neuroimaging Biomarkers for Predicting Treatment Response and Recurrence of Major Depressive Disorder

**DOI:** 10.3390/ijms21062148

**Published:** 2020-03-20

**Authors:** Seung-Gul Kang, Seo-Eun Cho

**Affiliations:** Department of Psychiatry, Gil Medical Center and Gachon University College of Medicine, Incheon 21565, Korea; arztin01@hanmail.net

**Keywords:** biomarker, major depressive disorder, genetics, neuroimaging, recurrence, treatment response

## Abstract

The acute treatment duration for major depressive disorder (MDD) is 8 weeks or more. Treatment of patients with MDD without predictors of treatment response and future recurrence presents challenges and clinical problems to patients and physicians. Recently, many neuroimaging studies have been published on biomarkers for treatment response and recurrence of MDD using various methods such as brain volumetric magnetic resonance imaging (MRI), functional MRI (resting-state and affective tasks), diffusion tensor imaging, magnetic resonance spectroscopy, near-infrared spectroscopy, and molecular imaging (i.e., positron emission tomography and single photon emission computed tomography). The results have been inconsistent, and we hypothesize that this could be due to small sample size; different study design, including eligibility criteria; and differences in the imaging and analysis techniques. In the future, we suggest a more sophisticated research design, larger sample size, and a more comprehensive integration including genetics to establish biomarkers for the prediction of treatment response and recurrence of MDD.

## 1. Necessity of Predictors for Treatment Response and Recurrence

Major depressive disorder (MDD) is the leading cause of disability contributing to the overall burden of disease globally. It is characterized by symptoms such as depressed mood lasting more than 2 weeks and causes emotional distress, functional impairment, health problems, and suicide, among others. MDD typically has a relatively good response to antidepressants; however, many patients fail to respond. The response rate to initial administration of antidepressants is cited to be approximately 50% in clinical trials [1]. Clinical guidelines recommend 4–8 weeks of treatment before considering an alternate medication in nonresponding patients [2]. The guidelines recommend that, if the treatment is ineffective after 1–2 months, a new medication or treatment should commence. Therefore, pharmacotherapy with unpredictable therapeutic response may cause persistence of depressive symptoms and functional impairment. If the patient reaches remission, duration maintenance therapy is usually recommended for 6 months to 1 year, but there are no predictors for recurrence after the discontinuation of antidepressants.

Recently, interest in precision medicine has increased. Clinicians have expressed interest in predicting which medications would be most effective and have the least adverse effects in specific patients or which non-pharmacologic treatments would be most effective. Thus, there have been studies of clinical and genomic predictors and biomarkers for treatment response and recurrence. In the last 20 years, hundreds of studies of neuroimaging biomarkers have been published. Neuroimaging techniques, such as magnetic resonance imaging (MRI), are intuitive tools that allow the structure and function of the brain to be visualized, and this is especially helpful in understanding psychiatric disorders and treatment response without invasiveness. The present review aims to describe current neuroimaging biomarkers for MDD.

## 2. Clinical and Genetic Predictors

Prior to the study of neuroimaging predictors, research was focused on clinical predictors of treatment response or recurrence. Subthreshold, inter-episodic depressive symptoms are associated with a shorter asymptomatic period and increased relapse and recurrence, which could cause more social and occupational dysfunction [3]. Clinical predictors of recurrence of MDD include early-onset [4], greater familial risk [5], and family history of mental disorders other than MDD (i.e., substance use disorders, anxiety disorders, antisocial personality disorder) [6,7,8].

Research has also sought to determine whether the physiological characteristics of sleep in MDD could be predictors for recurrence, since MDD is closely related with sleep deficits and changes in sleep architecture. In MDD, polysomnography results show decreased slow wave sleep and disturbed regulation of rapid eye movement (REM) sleep (i.e., decreased REM latency and increased REM sleep duration and density). These polysomnography findings have been considered neurophysiological markers of MDD that might estimate future recurrence or relapse [9]. In adults aged 60 years or older, persistent sleep disturbances have been associated with increased risk of the recurrence and worsening of depression [10]. Patients with MDD and insomnia who had evening circadian preference were at an increased risk for poor treatment response to pharmacological treatment and cognitive behavioral therapy (CBT) for insomnia or control therapy [11].

Due to previous studies investigating the heritability of MDD from family and twin studies, efforts to identify specific genes associated with disease onset, treatment response, and recurrence of MDD have begun. From previous studies, the heritability of MDD has been estimated as 37% [12], with evidence showing that recurrent MDD demonstrates greater familial risk [13]. The United States Food and Drug Administration and the European Medicine Agency started including pharmacogenetic information regarding pharmacokinetic (e.g., cytochrome P450 2D6 and 2C19) and pharmacodynamic (e.g., brain-derived neurotrophic factor (BDNF), 5-hydroxytryptamine receptor 2A, and G protein subunit beta 3) processes in the labeling of antidepressants [14]. Understanding the association between the treatment response of MDD and genotypes may be critical for personalized therapy; however, the clinical utility and cost-effectiveness of pharmacogenomic testing is not yet supported by replicated evidence [15]. Additionally, recent genome-wide association studies on recurrent MDD have failed to identify genetic polymorphisms that reach genome-wide significance [16,17].

## 3. Neuroimaging Biomarkers

With advances in neuroimaging techniques, biomarkers from neuroimaging studies are important in achieving precision medicine for many psychiatric disorders. Recently, the identification of neuroimaging predictors (recently called moderators) throughout the course of MDD has become an important research topic of the National Institute of Mental Health and pharmaceutical industry [18,19]. Neuroimaging methods to find moderators for treatment response include brain volumetric MRI, functional MRI (fMRI; resting state and affective tasks), electroencephalography (EEG), diffusion tensor imaging (DTI), magnetic resonance spectroscopy (MRS), near-infrared spectroscopy (NIRS), and molecular imaging (i.e., positron emission tomography (PET) and single photon emission computed tomography (SPECT)). These imaging techniques have been used to predict treatment response or recurrence of MDD, depending on the aim and nature of the study.

### 3.1. Structural Imaging and Volumetric Data

Neuroimaging studies use diverse techniques to image the structure, function, or molecular neuropharmacology of the brain. MRI scans use magnetic fields to discriminate among gray matter, white matter, and cerebrospinal fluid. Recently, 3T MRI has been widely used for neuroimaging research in humans; however, 7T MRI is also being started to be used for higher resolution. Additionally, 11.74T and 14T MRIs are being developed, and in the future, 20T MRIs will hopefully be developed and used [20].

Many structural brain imaging studies have been conducted on treatment response and recurrence. The majority of previous studies have found that specific area of brain volumes before treatment initiation had predictive potential for drug response outcomes. Smaller brain volumes typically estimate poorer treatment response, whereas larger brain volumes correlate with good response and remission [21].

The hippocampus has been reported to be important in the etiology of MDD [22,23,24]. Elevated levels of glucocorticoids in MDD have been hypothesized to be related to damage to the hippocampus, which is a brain region involved in memory and learning. Patients with MDD typically show a smaller (by 19%) left hippocampal volume than healthy controls [22]. The hippocampus is also the most frequently reported region when studying the association between volume and treatment response [25,26,27,28,29,30,31]. Volumetric changes of the hippocampus and amygdala were measured in participants with MDD and control participants between baseline and 1-year follow-up scans [25]. The non-remitters after treatment follow-up had significantly decreased brain volumes of the bilateral hippocampus compared to remitters [25]. A meta-analysis and systematic review of the research on treatment response predictors using MRI summarized that the decreased right hippocampal volume was a significant estimator of poor treatment response [27]. The comparison of regional (head and body/tail) hippocampal volumes between MDD remitters and non-remitters showed that the remitted patients had larger pretreatment hippocampal body/tail volumes [30].

In addition, various brain regions are reported to be involved in treatment response. A voxel-based morphometry study of 50 MDD patients reported that a smaller right lingual gyrus prior to treatment predicted poorer response [32]. Brain structure before treatment has been reported to predict clinical remission to the antidepressant fluoxetine with 88.9% accuracy, and the gray matter volumes in the middle frontal gyrus, occipital lobe, and cingulate cortex in remitters were larger than in non-remitters [33]. A multivariate pattern analysis using the gray matter and white matter magnetic resonance image at baseline differentiated treatment-sensitive patients from treatment-resistant patients with 82.9% accuracy [34]. A large, multisite, and placebo-controlled MRI study examined the association between the efficacy of sertraline and the cortical thickness at baseline and after 1 week [35]. It was found that increased cortical thickness of the rostral anterior cingulate cortex after 1 week of treatment was associated with better response to selective serotonin reuptake inhibitors (SSRI) at 8 weeks; however, the predictive accuracy (63.9%) in differentiating treatment response from nonresponse was relatively limited [35]. The structural MRI results of 53 unipolar depressed patients showed that a larger amygdala volume was significantly associated with a lower post- electroconvulsive therapy (ECT) Montgomery–Asberg Depression Rating Scale score. [36] A voxel-based morphometry (VBM) study in 55 patients with MDD and 23 healthy controls suggested that a smaller volume of the inferior frontal gyrus at baseline was related to faster treatment response while a smaller volume of the right lateral temporal cortex pretreatment was related to a greater response to ECT [37]. A systematic review of neuroimaging studies found the strongest evidence for increased volume within the temporal lobe and subcortical structures in responders to electroconvulsive therapy (ECT) and a significant association between higher pretreatment anterior cingulate cortex volume and the response to antidepressants, ECT, and CBT [38]; however, only four of 11 studies reported a significant association between the results of the changes in gray matter volume and the response to antidepressants [38]. A recent naturalistic 5-year follow-up study found that the addition of structural MRI findings to clinical information in MDD significantly improved clinical outcome predictions [39] and that the dorsal portion of the right anterior cingulate gyrus was an important predictor for long-term clinical outcomes [39].

Associations between white matter hyperintensities and low therapeutic responses have also been reported. Subcortical white matter hyperintensities in the left hemisphere have been associated with low therapeutic response and low remission to fluoxetine [40,41], and an increased number of white-matter hyperintensities was also correlated with the response to untreated late life depression [42,43]. It has also been reported that increased white matter hyperintensities are associated with non-remission to SSRI and serotonin-norepinephrine reuptake inhibitor (SNRI) treatment [44].

### 3.2. Diffusion Tensor Imaging

DTI is a neuroimaging technique that visualizes white matter neural tracks using the restricted diffusion of water in brain tissue. Diffusion MRI and DTI methods have led to the study of white-matter connectivity and abnormalities in conventional brain MRI of white matter. DTI has been used to study the disruption of neural connectivity in many psychiatric disorders, such as schizophrenia [45]. The most commonly used measures in DTI include fractional anisotropy (FA) and mean diffusivity (MD) [45].

In a previous study using electronic medical records from multiple sites, non-remitters had lower FA value on DTI measures in the medial fornix than control subjects [46]. In addition, MDD non-remitters to sertraline had higher FA values in the superior frontal and anterior cingulate cortices [47]. The relationship between the 1-year change in DTI measures and the 1-year course of MDD was examined, and it was found that non-remitted depressed patients showed significantly less change (i.e., reduction) in FA of the anterior cingulate cortex than remitted depressed and healthy control participants [48]. It was also reported that remitted MDD patients demonstrated increased FA and decreased MD in the left amygdala despite comparable amygdala volumes [49]. A preliminary study that investigated the relationship between treatment response to ketamine and white-matter connectivity using Track-Based Spatial Statistics (TBSS) method found significant differences in FA, radial diffusivity (RD), and MD between responders and nonresponders in frontolimbic and ventral striatal pathways [50]. Another DTI study of 102 MDD outpatients showed that connectivity for the anterior cingulate-limbic white matter significantly predicted remission with 62% accuracy, independent of demographic and clinical information, and with 74% accuracy when age was included in this model [51]. The post-ECT amelioration of symptoms was causatively associated with a decrease in RD in the dorsal frontolimbic circuits and decreased RD and FA levels in the anterior thalamic radiation and left anterior cingulum [52]. Studies have also reported that DTI-derived metrics (i.e., FA ratio of the stria terminalis over the cingulate portion of the cingulate bundle) identified non-remitting participants with an accuracy of 86% [53]. A final DTI study that examined biological predictors of SSRI response reported greater FA between the amygdala and raphe nucleus in non-remitted patients than in those who were remitted [54].

### 3.3. Functional MRI

fMRI measures the signal elicited from blood flow changes in the brain that are associated with neural activity. Functional magnetic resonance images reflect activated brain regions during resting state or task performance. The primary advantage of fMRI over other imaging techniques is its noninvasiveness, which allows for participants to not be exposed to ionizing radiation or radiopharmaceuticals, unlike PET and SPECT [55].

fMRI studies of treatment response have largely consisted of resting state or task-based (cognitive or affective) fMRI. Resting state fMRI studies are considered to reflect traits more than task-based fMRI studies. In resting state fMRI, low pretreatment resting state functional connectivity (RSFC) within the cognitive control network has been reported to be associated with non-remission to escitalopram in late life MDD [56]. Contrarily, reduced baseline RSFC within the default mode network of the orbitofrontal component predicted clinical response to duloxetine [28]. The association between increased RSFC in the striatum and treatment resistance to both SSRIs and SNRIs was reported [57]. Treatment response was associated with high RSFC between the amygdala and right medial and middle frontal cortices; however, it was also associated with low RSFC between the amygdala, right posterior cingulate cortex, and right precuneus [58]. Therefore, these results are largely inconsistent among studies. A systematic review of the relationship between treatment response in MDD and resting state fMRI also reported that direct comparisons across studies were difficult since there was considerable variability in study designs and analytic methods (i.e., regional homogeneity, seed-based analyses, and independent component analysis) [59]. The review summarized previous results as follows: (1) significant associations between treatment response to antidepressants and high functional connectivity between the frontal and limbic areas likely due to greater inhibitory control over the neural circuits for emotional process; (2) a low connectivity of visual recognition circuits in nonresponding MDD patients than responding patients; and (3) consistent findings of the association between response to repetitive transcranial magnetic stimulation (rTMS) and subcallosal cortex connectivity [59].

Task-based fMRI studies have also investigated the association between treatment response and brain activation with task performance. Brain activation in the bilateral inferior frontal cortex, insula, nucleus accumbens, and left amygdala, during successful inhibitory events of the parametric go/no-go task and activation in the rostral anterior cingulate during unsuccessful inhibition (commission errors) predicted posttreatment improvement of depression [60]. Outcomes of the fMRI study during the response inhibition (Go/No-Go) task in MDD patients treated with SSRI (escitalopram and sertraline) and venlafaxine showed that the dorsolateral prefrontal cortex generated a greater response during the cognitive task at baseline in the subjects with remission than in those without remission [61]. However, the SSRI and SNRI medications showed different activation patterns in the inferior parietal area [61]. The SSRI remitters showed a greater activation level at baseline than the non-remitters; however, the SNRI remitters showed the opposite patterns [61]. An Australian study subjected 26 patients with treatment-resistant MDD to fMRI analysis during the planning task before and after r-TMS treatment. Those that responded to low-frequency rTMS treatment showed a bilaterally decreased activation in the middle frontal gyrus [62].

In addition to cognitive tasks, fMRI studies have included tasks to involve the participants’ emotional reactions or rewards processing. A preliminary study in 17 unmedicated MDD participants reported that lower pretreatment blood oxygen level-dependent signals to negative words in the midbrain, anterior cingulate, paracingulate, dorsolateral prefrontal cortex, thalamus, and caudate nuclei were significantly correlated with a greater reduction in depressive symptoms after escitalopram treatment [63]. The activity in the amygdala to negative faces was significantly lower in treatment responders versus nonresponders to paroxetine treatment [64]. The fMRI scanning during the emotional face in the 24 MDD patients and 15 healthy controls was performed before and 4 weeks after administering venlafaxine or mirtazapine. Interestingly, a significant decrease in the blood-oxygen-level dependent (BOLD) signal responses during the treatment period was observed in the thalamus, basal ganglia, hippocampus, and cerebellum in the venlafaxine group and a significant increase in BOLD responses was observed in the supplementary motor area and middle cingulate gyrus in the mirtazapine group [65]. Larger changes in BOLD signal in the left fusiform gyrus at baseline was associated with a better treatment response to venlafaxine, while minor changes of the BOLD signal in the right rolandic operculum at baseline was related with a better response to mirtazapine [65]. The fMRI study on MDD patients treated with escitalopram, sertraline, and venlafaxine XR showed that a lower baseline amygdala activity to the subliminal happy and threat stimuli predicted the overall treatment response to antidepressants and that a higher baseline response to sad stimuli was related with nonresponse to venlafaxine [66]. The Establishing Moderators and Biosignatures of Antidepressant Response in Clinical Care study examined whether reward-related neural activity, while performing a reward task, moderated response to the antidepressant sertraline, an SSRI that targets dopamine and serotonin [67]. The authors found a significant moderation effect of reward-related ventral striatal activity on antidepressant response [67].

### 3.4. Electroencephalography

Electroencephalography (EEG) has provided further understanding on the treatment response or recurrence in previous studies. Baseline gamma power (20–70 Hz) at frontal, central, and temporal electrodes before treatment with paroxetine was significantly related to treatment response in MDD patients [68]. The altered late gamma band activity to positive words was found to be a persistent biomarker of the risk of recurrent MDD [69]. Responders to antidepressants had significantly higher EEG-vigilance stage (stage with relaxed wakefulness) and lower EEG-vigilance stage (stage with drowsiness) than nonresponders [70]. In a previous EEG study using the source localization method (i.e., exact low-resolution brain electromagnetic tomography analysis), nonresponding MDD patients had higher theta waves in resting state EEG in the frontal and rostral anterior cingulate cortices [71]. In an additional recent study, machine learning models of EEG profiles and clinical features were predictive of the antidepressant response [72]; however, since the number of participants in some previous EEG studies is relatively small [68,69] and the results are inconsistent, EEG studies must be replicated using large independent samples in the future.

### 3.5. Molecular Imaging (PET and SPECT)

PET scan is a molecular imaging method that uses a radioactive tracer to display brain function. One of the biggest advantages of PET is that the use of the tracer in the initial phase of drug development is possible, and drug binding can be studied to find clinically relevant doses of medication [73]. SPECT is a tomographic imaging technique that uses gamma-emitting radioisotope with advantages such as good spatial resolution, high sensitivity, no need for cyclotron, relatively long half-life, and limitless penetration depth; therefore, SPECT has an important role in preclinical and clinical studies [74]. The pretreatment assessment of brain metabolism using molecular imaging methods such as SPECT and PET has predicted treatment response and remission to antidepressants or nonpharmacological interventions.

In a previous study, pretreatment regional brain glucose uptake in the thalamus, midbrain, and putamen on PET predicted remission [75]. Changes in brain glucose metabolism were measured using PET in fluoxetine-treated MDD patients, and clinical improvement was associated with a decrease in the responses of both the limbic and striatal regions and with increase in the brain stem and cingulate [76]. In the study, nonresponse was related with the absence of changes in the subgenual cingulate or prefrontal areas [76]. Baeken et al. found that a higher baseline glucose metabolism during PET scans using 18F-fluoro-2-deoxy-d-glucose radiotracers (18FDG-PET) of the anterior cingulate cortex was a marker of positive response to rTMS [77]. The nonresponders to venlafaxine treatment or CBT exhibited pretreatment hypermetabolism with 18FDG-PET at the interface of the subgenual and pregenual cingulate cortices [78]. Interestingly, McGrath’s study showed the possibility of treatment-specific molecular imaging biomarkers because insular metabolism was opposing, depending on the treatment type (i.e., escitalopram versus CBT) [79]. In McGrath’s study, hypometabolism in the insula on PET was associated with remission with CBT and nonresponse to escitalopram; however, hypermetabolism in the insula was associated with remission with escitalopram and nonresponse to CBT [79]. Another study had attempted to do the same in 17 MDD patients who were treated with CBT and examined using 18FDG-PET; furthermore, post hoc comparison to paroxetine-treated responders was also performed to compare the difference [80]. The treatment response to CBT resulted in increases in the dorsal cingulate and hippocampus and decreases in the frontal cortex, and this pattern was distinct from the that in the paroxetine-treated group (hippocampal and subgenual cingulate decreases and prefrontal increases) [80]. High serotonin transporter (SERT) binding at baseline in the striatum and thalamus was related to early improvement of symptoms, and low SERT binding in the striatum was related to poor antidepressant response [81]. In an 11C-ABP688 PET study in MDD, the reduction of metabotropic glutamatergic receptor availability by ketamine was associated with treatment response to antidepressants [82]. An additional PET study using dopamine D2/3 receptor-selective radiotracer 11C-raclopride studied the relationship between the availability of D2/3 receptors and treatment response to antidepressants [83]. The authors reported that non-remitted MDD participants showed greater D2/3 availability prior to treatment in the nucleus accumbens/ventral pallidum and greater dopamine release after placebo in the bilateral nucleus accumbens [83].

SPECT studies have been used to predict response to various treatment modalities (e.g., pharmacotherapy and nonpharmacologic therapies such as psychotherapy). Lower regional cerebral blood flow (rCBF) levels in the right middle frontal cortex in nonresponding MDD participants was compared to those in responders to SSRI treatment [84]. CBT responders showed a significant increase in SERT standardized uptake ratio values in the left and medial temporal lobe regions [85]. Patients with MDD who showed good treatment response to ECT also showed normalization of rCBF [86]. There was a significant correlation between the treatment response after rTMS and the ratio of rCBF in the dorsolateral prefrontal cortex to the ventromedial prefrontal cortex [87]. SPECT-guided rTMS in treatment resistant MDD has been found to have the potential to guide decisions of the regions for brain stimulation in rTMS [88]. The stimulation to the hypoperfused area in the left prefrontal cortex versus the left dorsolateral prefrontal cortex yielded greater improvements with rTMS [88].

### 3.6. Magnetic Resonance Spectroscopy and Near-Infrared Spectroscopy

MRS detects various molecular concentrations and provides quantitative biochemical information of the tissues. It allows viewing of brain chemical activity rather than high-resolution images, while MRI creates anatomical images. A 3T proton MRS study showed that responders to rTMS had lower baseline concentrations of glutamate in the dorsolateral prefrontal cortex compared to nonresponders [89]. A naturalistic, open-label treatment study of rTMS investigated the gamma-aminobutyric acid (GABA) and glutamine levels before and after treatment using 1H MRS [90]. GABA levels in the medial prefrontal cortex increased after rTMS treatment, and this increase was greater in responders than in nonresponders [90]. The proton MRS study in treatment-resistant MDD investigated prefrontal cortex before and after rTMS, and it found the significant positive correlation between myo-inositol metabolism change and clinical improvement of depression [91].

NIRS is a neuroimaging technique that measures changes in the concentration of oxygenated hemoglobin or deoxygenated hemoglobin in biological tissue. It is a noninvasive method and reduces discomfort to participants; therefore, large sample sizes using this method is more achievable. In a multi-site NIRS study, hemodynamic changes in the prefrontal area during arithmetic and drawing tasks before rTMS treatment were evaluated [92], and low hemodynamic response during a cognitive task at the stimulation site significantly predicted the clinical response to active rTMS [92]. A 12-week longitudinal study explored the hemodynamic changes of the frontal area every 4 weeks using 52-channel NIRS and found a significant negative correlation between mean oxygenated hemoglobin values after 4 weeks and changes in total score of Hamilton Rating Scale for Depression from 4 to 8 weeks and 4 to 12 weeks [93]. A longitudinal NIRS study in medication-naïve MDD showed a significant association between mean oxygenated hemoglobin values during a verbal fluency task in the middle temporal and bilateral inferior frontal regions before antidepressant treatment and improvement of depression following antidepressant treatment [94]. The correlation analysis found that the NIRS signals during a cognitive task before the initiation of antidepressants could be a biological marker to predict treatment response [94].

### 3.7. Imaging Pharmacogenetics

Integrating neuroimaging findings and other biological findings such as genetic, epigenetic, and metabolomic markers can improve the predictive power for treatment response in clinical situations. A representative example is the Enhancing NeuroImaging Genetics through Meta-Analysis Consortium, which integrates neuroimaging and genetic data [95]. Previous imaging pharmacogenetic studies have examined the serotonin transporter-linked polymorphic region (5-HTTLPR) polymorphism and found that amygdala activity changes during emotional task fMRI (response to negative emotional face stimuli) after treatment were greater in LL homozygotes than S allele carriers of 5-HTTLPR polymorphism [96]. The endocannabinoid system has been considered to be involved in the pathogenesis of MDD. Non-remission to antidepressant treatment was associated with the G-allele of the rs1049353 polymorphism of the cannabinoid receptor 1 gene. Additionally, G-allele carriers showed attenuated brain activation in the putamen, thalamus, and amygdala during fMRI in response to masked happy facial stimuli [97]. The imaging pharmacogenetic study that had examined the BDNF gene Val66Met polymorphism and VBM revealed a significantly negative correlation between the left hippocampal volume and the days required to remission in Val66 homozygotes of the BDNF gene Val66Met polymorphism [98]. The DTI study suggested that depressed patients who were BDNF Met carriers had a higher remission rate and microstructural abnormalities expressed as FA values were associated with a lower remission rate [99]. However, there was no significant interaction between the BDNF Val66Met polymorphism and DTI findings [99]. An imaging genetic study that investigated DTI-TBSS and polymorphisms (BDNF Val66Met and 5-HTTLPR) found that the predictive value of white matter connectivity in antidepressant response may be influenced by genetic polymorphisms in MDD [100]. In this previous study, the interaction between 5-HTTLPR polymorphisms and FA value in the right uncinate fasciculus predicted the change in MDD severity [100]. Recently, the machine learning model called the ensemble learning prediction model showed improvements in sensitivity and accuracy of prediction after integrating imaging and genetic data [101]. Compared to the single-level prediction method, the ensemble learning prediction model using support vector machines utilized 14 brain regions of interest and integrated genetic features [101].

### 3.8. Machine Learning

There are several neuroimaging and imaging meta-analytic studies regarding the etiology and treatment response of mood disorders; however, it is difficult to identify the disease or to predict its response in clinical settings via brain imaging results alone. For example, since neuroimaging studies typically analyze group differences, some patients with MDD demonstrate a similar volume to that of the healthy controls in specific regions of interest in the brain. However, our patient cannot be declared healthy by using only MRI findings [102]. Machine learning can help solve these problems, as studies in this field characteristically investigate an individual-level comparison. Machine learning techniques can be used to predict the disease, to classify the diagnoses, and to predict the treatment response. Most machine learning studies on mood disorders were performed to elucidate the diagnostic classification [33,103,104,105] and treatment response [34,106,107]. Neuroimaging biomarkers are advantageous in being considerably more stable than peripheral biomarkers, and they are not affected by inflammation or nutrition. However, data sharing and multi-site participation are considered necessary due to the difficulty in achieving an adequate sample size from a single research site [102].

## 4. Current Status and Limitations of Neuroimaging Research in MDD and Future Studies

Many neuroimaging studies have been conducted on the treatment response in MDD, but the results are inconsistent. The reason for the lack of confirmed biomarkers is the different study designs; diverse characteristics of MDD participants from study to study; and the diversities of imaging, analytic methods, and imaging machines.

MDD is a heterogeneous disease with a variety of phenotypes, including the combination of core MDD symptoms, presence of psychotic features, cognitive function, and comorbidity. For a more accurate prediction of treatment response and recurrence, neuroimaging studies need to be performed for each biological phenotype of MDD.

In addition, future studies should include comprehensive data, other than neuroimaging findings, to increase the accuracy of predictions for response and recurrence. The integration of clinical data; neuroimmunoendocrine factors such as serum cortisol and thyroid hormones; genetic data; and electrophysiologic data, such as sleep and waking EEG, would improve the accuracy and sensitivity of the prediction for treatment response and recurrence. For example, the Canadian Biomarker Integration Network in Depression (CAN-BIND) project was designed to integrate comprehensive neuroimaging, electrophysiological, molecular, and clinical data to better predict treatment outcomes to antidepressant monotherapy and adjunctive therapy [108]. CAN-BIND first used escitalopram (10–20 mg/day) as a first-line antidepressant and an add-on medication (aripiprazole; 2–10 mg/day) in case of poor response after 8 weeks [108]. It is desirable to unify the dose or type of medication and treatment modalities to treat MDD in a research setting. Increasing the number of subjects, integrating data from multiple studies, and developing newer analytical methods through large data cohorts and machine learning will also help to develop clinically reliable and useful biomarkers.

## Abbreviations

MDD	Major depressive disorder
MRI	Magnetic resonance imaging
REM	Rapid eye movement
CBT	Cognitive behavior therapy
BDNF	Brain-derived neurotrophic factor
EEG	Electroencephalography
fMRI	Functional magnetic resonance imaging
DTI	Diffusion tensor imaging
MRS	Magnetic resonance spectroscopy
NIRS	Near-infrared spectroscopy
PET	Positron emission tomography
SPECT	Single-photon emission computed tomography
SSRI	Selective serotonin reuptake inhibitor
ECT	Electroconvulsive therapy
SNRI	Serotonin–norepinephrine reuptake inhibitor
FA	Fractional anisotropy
MD	Mean diffusivity
TBSS	Track-Based Spatial Statistics
RD	Radial diffusivity
RSFC	Resting state functional connectivity
rTMS	Repetitive transcranial magnetic stimulation
18FDG-PET	(18)F-fluoro-2-deoxy-d-glucose PET
SERT	Serotonin transporter
rCBF	Regional cerebral blood flow
GABA	Gamma-aminobutyric acid
5-HTTLPR	Serotonin transporter-linked promoter region
CAN-BIND	Canadian Biomarker Integration Network in Depression
